# Seeking the unseen: a review of host-finding strategies of parasitoids attacking wood-boring beetles

**DOI:** 10.1093/aesa/saag010

**Published:** 2026-04-10

**Authors:** Kelly L F Oten, Jian J Duan, Courtney L Johnson

**Affiliations:** Department of Forestry and Environmental Resources, North Carolina State University, Raleigh, NC, USA; USDA-ARS Beneficial Insects Introduction Research Unit, Newark, DE, USA; Department of Forestry and Environmental Resources, North Carolina State University, Raleigh, NC, USA

**Keywords:** biological control, Buprestidae, Cerambycidae, Curculionidae, host finding

## Abstract

The success of biological control of insect pests by parasitoids ultimately depends on effective host foraging by the agents, which involves multiple processes and various cues associated with hosts’ habitat, host-associated symbionts and other microbial organisms, and/or hosts themselves. In general, parasitoid interactions with concealed hosts such as wood-boring beetles differ significantly from those that attack exposed hosts (eg surface-feeding caterpillars, aphids) as they must rely on a more complex hierarchy of cues to first locate hosts’ microhabitats and then hosts for their reproduction. These differences influence the parasitoid’s host-finding strategies, specificity, and their efficacy in suppressing their host populations. Here, we review the major steps in host finding by parasitoids of wood-boring beetles and synthesize current knowledge on the chemical, vibrational, visual, and other cues that mediate each stage of the process. We highlight how these cues operate across different spatial scales, interact with parasitoid morphology and behavior, and shape the success of biological control programs targeting economically important wood-boring pests. Finally, we identify key knowledges gaps and point to some future research directions aimed at improving the selection, deployment, and monitoring of parasitoid biological control agents in forest ecosystems.

## Introduction

Wood-boring insect pests are primarily in the order Coleoptera (beetles), with fewer in Lepidoptera (moths) and Hymenoptera (horntails and sawflies), and a few cases in Diptera (flies). The most damaging species are beetles within Curculionidae (subfamily Scolytinae and Platypodinae), Buprestidae, and Cerambycidae, which develop beneath the bark or within woody tissues, remaining concealed throughout their immature stages. This cryptic nature poses unique challenges in detection, prevention, and insecticide-based management.

Nonnative wood borers have been especially consequential in the United States. Of the nonnative species entering the United States from 1860 to 2006, wood borers were among the most numerous and diverse, comprising about a quarter of the species deemed to have a high impact on trees (Aukema et al. 2010). Two of the most destructive invasive pests in North America in recent history are wood borers: the emerald ash borer, *Agrilus planipennis* Fairmaire (Coleoptera: Buprestidae), and the Asian longhorned beetle, *Anoplophora glabripennis* Motschulsky (Coleoptera: Cerambycidae). Since their accidental introductions, these beetles have caused extensive mortality of urban and forest trees, fundamentally altering forest composition and imposing substantial economic costs for tree removal, replacement, and long-term management. Emerald ash borer attacks and kills ash (*Fraxinus* spp.) while Asian longhorned beetle attacks a wide range of hardwood trees, with a strong preference for maple (*Acer* spp.) (Haack et al. 2010, Herms and McCullough 2014). Both species feed within host tree tissues, disrupting the translocation of water and nutrients and, in the case of Asian longhorned beetle, compromising their host tree’s structural integrity. Managing these two invasive beetles with chemical control strategies has proven challenging largely because of the impracticality and environmental concerns over the use of insecticides in natural forest settings. Thus, biological control with host-specific natural enemies may be a viable option for managing these invasive beetles, particularly if they can locate and attack insects concealed within host tissues to provide sustained, landscape-scale suppression in forest systems where chemical control is impractical.

Among the different types of biological control agents, parasitoids are generally regarded as the most useful due to their high efficacy and host specialization (Stiling and Cornelissen 2005, Clarke et al. 2019). Parasitoids are organisms whose immature stages feed and develop in or on a single host individual, ultimately killing it (Godfray 1994, Quicke 1997, Johnson 2013). This is a key distinction from true parasites, which typically do not kill their hosts (Godfray 1994). Most parasitoids occur within the order Hymenoptera, although notable lineages also exist in Diptera (Eggleton and Belshaw 1992, Johnson 2013) and Coleoptera (Wang et al. 2016).

Parasitoids are highly diverse in their biology and host use, yet only a small fraction are known to attack wood-boring insects, likely because the concealed development of these hosts within woody tissues reduces their accessibility compared with free-feeding insects (Dahlsten 1982, Godfray 1994, Hawkins et al. 1997). Along with endophytic gall formers and root feeders, wood borers experience some of the lowest levels of parasitoid-induced mortality (Hawkins et al. 1997). The most effective parasitoids of wood-boring beetles are within Hymenoptera and span numerous families, including but not limited to Ichneumonidae, Braconidae, Pteromalidae, Eupelmidae, Eulophidae, Eurytomidae, and Bethylidae (Wang et al. 2021).

Most of the parasitoids attacking bark or wood-boring beetles are larval ectoparasitoids and are typically idiobionts, arresting host development at or shortly after parasitism (Wegensteiner et al. 2015, Wang et al. 2021). The concealed habitat of wood borers favors ectoparasitism, as females can oviposit externally onto hosts. Endoparasitism is less common because oviposition would require penetrating the host body, which is far more difficult when hosts are concealed within wood (Godfray 1994, Quicke 1997). Some solitary parasitoids with shorter ovipositors (eg eurytomids and some braconids) generally target early instars located in or near the phloem, whereas species with longer ovipositors, which are often larger and gregarious, can reach later instars of borers that tunnel deeper into the xylem (Wang et al. 2021). Regardless, all parasitoids exploit suites of cues associated with their hosts’ habitats (location) and/or the host themselves to find suitable hosts for their reproduction. Often, these host finding processes involve multiple steps including habitat finding, host location, host discrimination (quality assessment), and acceptance (successful parasitism), which are all crucial for biological control success.

Given the small size of both parasitoid and host, along with the structural complexity of their environment, host location occurs under substantial spatial and sensory constraints (Godfray 1994). As biological control using parasitoids increasingly becomes a central strategy for managing destructive invasive pests, the ability of parasitoids to locate hosts hidden beneath bark or within wood emerges as a critical but poorly understood step in determining control success. Understanding how parasitoids overcome this formidable challenge is therefore essential for improving biological control programs targeting wood-boring pests.

In general, host finding by parasitoids of exposed hosts, such as surface-feeding caterpillars or aphids, differs greatly from host finding by parasitoids of concealed, wood-boring insects. Parasitoids attacking free-living, exposed hosts have access to a wide array of direct and reliable cues, many of which are unavailable when hosts develop cryptically within plant tissues. Most notably, hosts are visually apparent on plant surfaces, allowing some parasitoids to use visual information on host color (Michaud and Mackauer 1994, Fischer et al. 2004, Lobdell et al. 2005), size (Kouamé and Mackauer 1991, King 1994, Jenner and Kuhlmann 2006), and movement (Michaud and Mackauer 1994, Stireman 2002, Yamamoto et al. 2009), as well as conspicuous feeding damage (Wäckers 1994, Low 2008). Additionally, host-emitted chemical cues and herbivore-induced plant volatiles are often strong and closely associated with active feeding sites, enabling rapid host location (Godfray 1994, Aartsma et al. 2017). Hoballah and Turlings (2005) demonstrated that braconid parasitoids of maize-feeding caterpillars use herbivore-induced plant volatiles to locate hosts, with attraction to fresh versus older damage varying by species and prior oviposition experience. Other studies show parasitoids prefer volatiles from more heavily infested plants, suggesting that host abundance can be assessed indirectly through the quantity and composition of herbivore-induced plant volatiles (Girling et al. 2011, Song et al. 2021). Parasitoids can confirm host presence at close range through contact chemicals on the host cuticle detected during antennation, as shown for aphid parasitoids (Michaud and Mackauer 1994), and some species can even detect trace “chemical footprints” left behind by walking caterpillars for up to 2 d (Wölfling and Rostás 2009). Plants may also emit volatiles following insect egg deposition that attract egg parasitoids (Hilker and Meiners 2002, Peñaflor et al. 2011). In contrast, when hosts develop within wood, most visual cues are absent and chemical cues are often diluted, delayed, or physically separated from the feeding site (Quicke 1997, Johnson 2013, Fischbein et al. 2018). Therefore, parasitoids of wood-boring insects must rely on different and often less direct cues, which can make host finding more difficult than for parasitoids attacking exposed hosts.

These differences in cue availability have important implications not only for how hosts are detected but also for the morphological and behavioral mechanisms required for successful parasitism. Parasitoids that attack wood-boring insects have adaptations that allow them to locate and oviposit on hosts concealed within woody substrates in structurally complicated forest tree ecosystems. These adaptations, a suite of which evolved convergently, include elongated, toothed ovipositors, strong transverse ridges on the mesonotum, and chisel-like mandibles (Quicke 1997, Dal Pos and Sharanowski 2024). While these morphological adaptations are well established, the mechanisms, traits, and behaviors that parasitoids of wood-boring beetles use to locate their hosts remain relatively understudied (Johnson 2013).

Previous work has examined the stimuli parasitoids use to locate a wide range of hosts, but much of this research focuses on free-feeding or otherwise exposed hosts (Vinson 1976, Weseloh 1981, Vet and Dicke 1992, Steinberg et al. 1993, Godfray 1994, Vinson 1998, Giunti et al. 2015). Far less attention has been given to how parasitoids locate concealed wood-boring insects. Even within this group, most studies have focused on economically important bark beetles (*Ips* spp. and *Dendroctonus* spp.). However, research aimed at developing biological control programs for buprestids and cerambycids (primarily emerald ash borer and Asian longhorned beetle) has begun to incorporate investigations of host-location mechanisms underlying the parasitoid’s foraging efficiency, host specificity, and ability to establish populations to regulate the target pests. This review synthesizes the existing literature on how hymenopteran parasitoids locate scolytine, buprestid, and cerambycid wood-boring hosts, integrates insights from broader parasitoid–host systems, and identifies key knowledge gaps.

## Steps of Host Location

**Fig. 1.**
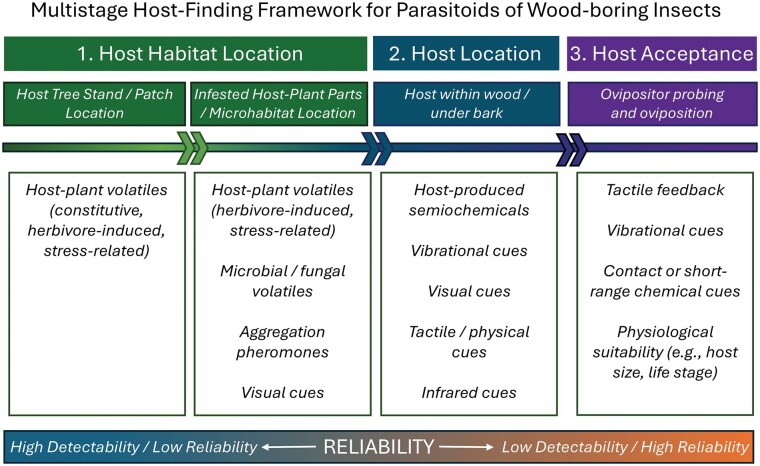
Conceptual framework illustrating the multistage host-finding process used by parasitoids attacking wood-boring insects. Parasitoids progress from long-range habitat and microhabitat location to short-range host localization and acceptance, relying on different sensory cues at each stage. Highly detectable but less reliable cues (eg host-plant volatiles) dominate early stages, whereas increasingly reliable but short-range cues (eg frass, vibrational, and tactile cues) are used to pinpoint hosts concealed within woody tissues.

To successfully reproduce, parasitoid wasps must navigate through a series of steps which are traditionally classified as host habitat location, host location, host discrimination or acceptance for parasitism, and host suitability for development (Fig. 1; Vinson 1976, Vet et al. 1995). For parasitoids that attack hosts concealed within plant tissues such as wood-boring beetles, the process often involves multiple, interacting stimuli associated with the hosts’ habitats and microhabitat or host-related microorganisms by foraging parasitoid adults (Vet and Dicke 1992, Fischbein et al. 2018).

When imaginal parasitoids emerge, they must first locate and navigate to their host’s habitat in order to reproduce. For the individual parasitoid, this is complicated by the fact that hosts are very rarely distributed evenly in the environment, instead occurring in discrete patches (Godfray 1994). In some systems, the ability of parasitoids to recognize patch boundaries and turn back to the patch has been demonstrated. For instance, both the parasitoid *Anagrus columbi* (Hymenoptera: Mymaridae) and its host, the planthopper *Prokelisia crocea* (Hemiptera: Delphacidae), exhibit boundary responses at habitat patch edges, often turning back into the patch (Cronin 2007). Among parasitoids of wood borers, *Ibalia leucospoides* (Hymenoptera: Ibaliidae), a parasitoid of wood wasps in the genus *Sirex* (Hymenoptera: Siricidae) can discriminate between different levels of host numbers among patches, preferentially choosing the patch with the highest number of hosts, even when surrounding habitat conditions are different. Authors of this study suggest that the parasitoid may obtain information from the habitat as a whole to make decisions about the use of patches, making it highly adaptable to patchy environments (Fischbein et al. 2012). Likewise, a study focusing on parasitoids of saproxylic beetles found that parasitoid wasps responded strongly to local habitat in a boreal forest, despite fragmentation (although the role of habitat area varied by parasitoid species; Gibb et al. 2008). The apparent ability of parasitoids to navigate patchy habitats is likely important for successful attacks on bark beetles; one study noted that typical infestations of *Dendroctonus frontalis* (Coleoptera: Curculionidae) consist of less than 10 trees, and these often decline, necessitating parasitoids to locate patchy resources (VanLaerhoven and Stephen 2002). Once a broader habitat or patch has been located, the parasitoid must then find the microhabitat (eg the host plant of the parasitoid’s host). Both habitat and microhabitat location often depend on long-range, volatile semiochemicals (Godfray 1994, Vet et al. 1995, Wäschke et al. 2013).

Once the host plant is reached, parasitoids must then locate their host. The process of locating a host is complex and involves a variety of cues, including visual, chemical, tactile, and vibrational or acoustic stimuli. Responses to these stimuli may be mediated by a complex of genetics, phenotypic plasticity, and learning behavior (Vet and Dicke 1992). Similar to their habitat finding behaviors, parasitoids may turn back to locate their hosts. In leafminer systems, for example, parasitoids can locate the host by orienting to mines and may reverse direction during search to remain on the mine and reach the concealed larva (Casas 1988, Godfray 1994, Ayabe and Ueno 2004). While the initial habitat location is typically learned, host detection and acceptance are innate, often made apparent by morphological adaptations (Vet et al. 1995, Laurenne et al. 2009, Dal Pos and Sharanowski 2024). After they find hosts, parasitoids must confirm host identity and suitability (size, life stage, etc.) before deciding to oviposit (Quicke 1997). Interestingly, concealment may play a role in host acceptance, particularly for ectoparasitoids. Ueno (2000) examined host selection of an ichneumonid wasp on its pyralid host, finding that while the wasps attacked both cocooned and exposed hosts, they discriminated between the 2 after ovipositor insertion, preferentially ovipositing on those which were concealed. Additionally, offspring fitness (survival and size) was higher within concealed hosts. A preference for concealed versus exposed hosts has likewise been noted in parasitoids of wood-boring beetles (eg Wang et al. 2010a) and stem borers (eg Rojas et al. 2006).

These steps highlight that successful host location by parasitoids is a multistage process that depends on the integration of multiple sensory cues operating across varied spatial scales. Understanding the cues that guide parasitoids during these stages is therefore critical for the effective development and implementation of biological control programs.

## Stimuli Used in Location of Wood-Boring Beetles

Despite the challenges posed by the concealed habitat of wood-boring beetles, research has identified a range of stimuli used by parasitoids of economically important wood-boring pests. Most studies have focused on chemical cues that facilitate host detection beneath bark, although visual and tactile cues have also been documented (Table 1). The sections below examine these stimuli, highlighting how different cue types contribute to host finding or location across parasitoid taxa and wood-boring beetle systems.

**Table 1.** Studies focusing on hymenopteran parasitoid host location of coleopteran wood borers with identification of the stimuli that they evaluated

References	Parasitoid wasp species	Bark- or wood-boring beetle host species	Type of stimuli used	Host-searching stage	Observed patterns/notes
**Boone et al. 2008**	*Heydenia unica*, *Rhopalicus pulchripennis*, *Dibrachys cavus*	*Ips pini*	Chemical	Host habitat location	Specialists responded to fungal symbiont cues whereas generalists used plant-derived volatiles
**Boone et al. 2009**	An assemblage of species including braconids, platygastrids, encrytids, pteromalids, and ichneumonids	*Ips pini*	Chemical	Host habitat location	Beetle- and microbe-colonized logs attracted a diverse assemblage of parasitoids, many of which were not bark beetle specialists, indicating use of shared habitat-level olfactory cues
**Broad and Quicke 2000**	Many species within Orussidae and Ichneumonidae	N/A	Vibrational	Host location	Used primarily to locate deeply concealed, immobile hosts, often in idiobiont parasitoids
**Camors and Payne 1972**	*Heydenia unica*	*Dendroctonus frontalis*	Chemical	Host habitat location	Attraction was strongest to host-tree terpene (α-pinene); beetle pheromones alone elicited weak or no attraction
**Camors and Payne 1973**	*Coeloides pissodis, Dendrosoter sulcatus, Spathius pallidus, Cecidostiba dendroctoni, Heydenia unica, Roptrocerus xylophagorum*	*Dendroctonus frontalis*	Chemical	Host habitat location	Predators arrived early during adult and egg stages, whereas parasitoids increased later during larval stages, suggesting stage-specific responses to beetle- and host-associated odors
**Hanks et al. 2001**	*Syngaster lepidus, Callibracon limbatus*	*Phoracantha semipunctata*	Vibrational	Host location	Parasitoids located hosts by vibration; species and sexes partitioned hosts by larval size and bark thickness, with host accessibility constrained by bark depth
**Hilszczański 2018**	*Doryctes leucogaster*	*Hylotrupes bajulus*	Chemical, vibrational, and tactile	Host habitat location, host location	Parasitoids show shared adaptations for finding hosts in dead wood, with species distributions linked to forest stage and wood characteristics
**Johnson et al. 2014**	*Spathius agrili, Spathius floridanus*	*Agrilus planipennis*	Chemical	Host location	*S. agrili* (a specialist) used host-plant cues and showed increased searching; *S. floridanus* (a generalist) responded only to infested stems
**Joyce et al. 2011**	*Syngaster lepidus*	*Phoracantha semipunctata, Phoracantha recurva*	Vibrational	Host location	Used vibrations from actively feeding larvae beneath the bark
**Kennedy 1979, Kennedy 1984**	*Cheirospachus colon, Entedon leucogramma, Dendrosoter protuberans, Spathius benefactor, Cerocephala eccoptogastri*	*Scolytus multistriatus*	Chemical	Host habitat location	Attracted to bark beetle pheromone components (strength and response varied by species)
**Lanier et al. 1972**	*Tomicobia tibialis*	*Ips pini*	Chemical	Host habitat location	Predators and parasitoids responded differently to pheromones produced by beetles from different regions
**Laurenne et al. 2009**	Several species in Ichneumonidae	Several species in Cerambycidae and Buprestidae	Vibrational	Host location	Antennal modifications for vibrational sounding were strongly associated with parasitism of deeply concealed, wood-boring hosts
**Li et al. 2015**	*Sclerodermus guani*	*Monochamus alternatus*	Chemical	Host location	Females responded to larval volatiles, with attraction varying by instar and experience
**Masini et al. 2024**	*Sclerodermus cereicollis, Sclerodermus domesticus*	*Hylotrupes bajulus, Trichoferus holosericeus*	Chemical	Host habitat location, host location	Used wood- and frass-derived odors and host cuticular cues at close range
**Mills et al. 1991**	*Coeloides bostrychorum*, *Dendrosoter middendorffi*, *Rhopalicuis tutela*	*Ips typographus*	Chemical	Host location	Host location depended on short-range volatiles; heat and vibration cues were not used
**Pettersson et al. 2000**	*Roptrocerus xylophagorum*	*Dendroctonus frontalis, Ips grandicollis*	Chemical	Host location	Females responded to host–tree complex volatiles (particularly oxygenated monoterpenes)
**Pettersson 2001b, Pettersson et al. 2001b**	*Rhopalicus tutela*	*Ips typographus* and other coniferous bark beetles	Chemical	Host habitat location, host location	Females responded primarily to oxygenated monoterpenes from damaged host trees; heat and larval cues were not used
**Pettersson 2001a**	*Rhopalicus tutela, Roptrocerus mirus, Roptrocerus xylophagorum*	*Ips typographus*	Chemical	Host habitat location	Females responded to oxygenated monoterpenes from infested spruce; males showed weak or no attraction
**Pettersson et al. 2001a**	*Coeloides bostrichorum*	*Ips typographus*	Chemical	Host habitat location	Attraction driven by oxygenated monoterpenes associated with infested spruce
**Pettersson and Boland 2003**	N/A	*Ips typographus*	Chemical	Host habitat location	Volatile blends changed over the course of infestation; oxygenated monoterpenes and benzenoids increased as susceptible larvae developed; may explain stronger attraction to natural odors than synthetic baits
**Richerson and Borden 1972b**	*Coeloides brunneri*	*Dendroctonus pseudotsugae*	Heat/infrared radiation	Host location, host acceptance	Females oviposited directly over thermal “hotspots” associated with larvae; artificial heat sources alone elicited searching and oviposition
**Richerson and Borden 1972a**	*Coeloides brunneri*	*Dendroctonus pseudotsugae, Dendroctonus ponderosae*	N/A	Host location	Antennal amputation resulted in inability to locate host galleries; no evidence supported use of vibrational, magnetism, or chemical cues
**Ryan and Rudinsky 1962**	*Coeloides brunneri*	*Dendroctonus pseudotsugae*	Vibrational, visual	Host location, host acceptance	Host finding depended on larval feeding vibrations, with oviposition favored in shaded conditions
**Salom et al. 1991, 1992**	*Dinotiscus dendroctoni, Coeloides pissodis*	*Dendroctonus frontalis*	Chemical	Host habitat location, host location	Broad antennal sensitivity to multiple host- and beetle-derived volatiles, with some sex-specific thresholds
**Samson 1984**	*Roptrocerus xylophagorum*	*Ips grandicollis*	Tactile	Host location	Females entered bark beetle galleries and located hosts by antennal tapping
**Sullivan and Berisford 2004**	*Roptrocerus xylophagorum, Spathius pallidus*	*Ips* spp., *Dendroctonus frontalis*	Chemical	Host habitat location, host location	Fungal odors increased laboratory attraction, but were not required for host attack; field attraction required the full host–tree complex
**Sullivan et al. 1997**	*Spathius pallidus, Roptrocerus xylophagorum*	*Dendroctonus frontalis*	Chemical	Host habitat location	Attraction required blended bark volatiles; single compounds or simple synthetic blends were ineffective
**Sullivan et al. 2000**	*Roptrocerus xylophagorum*	*Ips grandicollis*	Chemical	Host habitat location, host location	Attraction required odors from infested bark and frass rather than hosts alone, indicating reliance on the full host–plant complex
**Ulyshen et al. 2011**	*Tetrastichus planipennisi*	*Agrilus planipennis*	Vibrational	Host acceptance	Vibration amplitude increased with host age/size, suggesting a role in host acceptance
**Wang et al. 2010a**	*Spathius agrili*	*Agrilus planipennis*	Chemical and Vibrational	Host habitat location, host location, host acceptance	Females used ash volatiles to locate host habitat, then relied on host-generated vibrations to locate and accept larvae; contact chemicals played little role, and females avoided already parasitized (nonvibrating) hosts
**Wei et al. 2013a**	*Dastarcus helophoroides*	*Anoplophora glabripennis, Apriona swainsoni*	Chemical	Host habitat location, host location	Parasitoid attraction differed by host species, with population-specific responses to frass-derived monoterpenes
**Wei et al. 2013b**	*Sclerodermus pupariae*	*Agrilus planipennis, Massicsu raddei*	Chemical	Host habitat location, host location	Prior experience altered olfactory preferences; parasitoids learned to associate novel host–plant–frass volatiles with hosts, resulting in increased attraction, paralysis efficiency, and parasitism of a nonnative host
**Yang et al. 2008**	*Spathius agrili*	*Agrilus planipennis*	Chemical	Host habitat location	Females were attracted only to volatiles from EAB-infested ash and not to other potential host plants

This is likely not a comprehensive list but points to the large focus on study of chemical cues and economically important host species.

### Chemical Cues

Parasitoids primarily locate hosts through chemical communication, using a complex hierarchy of cues collectively referred to as semiochemicals or infochemicals (Takabayashi 2022). These cues vary in source, reliability, and spatial scale and are often integrated sequentially during host finding. While some chemical signals originate directly from the host insect, many are produced by host plants or associated microorganisms, creating multitrophic interactions that are especially important for parasitoids attacking concealed hosts such as wood-boring beetles.

#### Host-Plant Volatiles

Host-plant volatiles play a central role in long-range host habitat and microhabitat location. Dietary specialization plays a role in this relationship, as generalist parasitoids are more likely to rely on plant-derived cues, whereas specialists are more likely to rely on host-derived semiochemicals such as pheromones (Vet and Dicke 1992, Boone et al. 2008, Masini et al. 2024). Numerous studies demonstrate the importance of host plant stress volatiles, either through direct behavioral assays or indirectly via increased parasitism in stressed trees (Pettersson et al. 2000, 2001a, b, Shibata 2000, Sullivan et al. 2000, Wang and Yang 2008, Dodds et al. 2023).

Host-plant volatiles are generally highly detectable over long distances but may be relatively unreliable indicators of host presence, resulting in the “reliability–detectability dilemma.” This concept proposes that stimuli must be both detectable from long distances (a difficult ask for concealed hosts) and reliable, in which they actually indicate host presence, 2 properties that are often inversely related (Fig. 1; Vet et al. 1991, 1995, Vet and Dicke 1992). For example, host-plant volatiles are readily detected but are relatively unreliable cues, as the presence of a host plant does not necessarily indicate that an appropriate host life stage is present (Vet et al. 1995). The reliability–detectability dilemma highlights that parasitoids must balance finding cues that are abundant and easy to detect with finding cues that are less abundant but indicate host presence, a trade-off that strongly influences their use of chemical information.

In some of these cases, chemical cues may include herbivore-induced plant volatiles that function as synonomes, signals that benefit both the host plant and the natural enemy. These volatiles are released by the host plant in response to herbivore feeding (Vet and Dicke 1992) and may include terpenoids such as α-pinene, d-limonene, 3-octanone, and 4,8-dimethyl-1,3,7-nonatriene (Turlings and Tumlinson 1992, Turlings and Erb 2018). Recent work further suggests that volatiles emitted by intact, uninfested plants may function as repellent or contrast cues, helping parasitoids discriminate infested patches from the surrounding environment (Ross et al. 2023). In conifer systems, oxygenated monoterpenes released by stressed trees following bark beetle attack are particularly important. Scolytine parasitoids such as *Roptrocerus xylophagorum* (Hymenoptera: Pteromalidae), *Rhopalicus tutela* (Hymenoptera: Pteromalidae), *Dinotiscus dendroctoni* (Hymenoptera: Pteromalidae), and *Coeloides bostrichorum* (Hymenoptera: Braconidae) respond strongly to these compounds (Salom et al. 1991, Pettersson et al. 2000, 2001a, b, Sullivan et al. 2000). These cues may also convey information about host developmental stage, as oxygenated monoterpenes and benzenoid compounds increase as bark beetle larvae develop (Pettersson and Boland 2003). Nevertheless, no single compound appears sufficient to explain parasitoid attraction; instead, mixtures of host-, plant-, and beetle-derived semiochemicals consistently outperform individual cues (Spradbery 1970, Sullivan et al. 1997, Boone et al. 2008, 2009, Johnson et al. 2014).

In wood-boring insect-parasitoid systems, host-plant volatiles appear particularly important for long-range orientation. For example, several larval parasitoids in the genus *Spathius* are attracted to volatiles emitted by ash bolts infested with EAB larvae (Wang et al. 2010, Johnson et al. 2014). However, the specific compounds responsible for attraction in these systems remain largely unidentified.

#### Host-Produced Volatiles and Byproducts

Although chemical cues originating directly from the host are often more reliable indicators of host presence, they are typically detectable only at shorter range. As parasitoids move from habitat location to host location, nonvolatile or weakly volatile cues produced by the host, such as frass, sawdust, or gallery-associated odors, become increasingly important (Vet and Dicke 1992). Because hosts experience strong selective pressure to reduce conspicuousness, parasitoids frequently rely on indirect host cues rather than signals emitted by larvae alone (Vinson 1998).

Early studies in bark beetle systems observed peak parasitoid attraction several days after beetle aggregation, suggesting responses to cues other than aggregation pheromones, including frass or induced plant responses (Berisford and Franklin 1971, Borden 1982). Similarly, parasitoids of cerambycids [*Sclerodermus cereicollis*, *S. domesticus* (Hymenoptera: Bethylidae)] and Sirex woodwasps [*Rhyssa persuasoria* (Hymenoptera: Ichneumonidae)] respond strongly to sawdust and frass rather than to larvae alone (Spradbery 1970, Masini et al. 2024). Studies on stem borer parasitoids in coffee systems further showed that host frass greatly increased attraction, whereas frass derived from artificial diets did not, implicating plant-mediated chemical responses (Potting et al. 1995, Rojas et al. 2006).

Often, host-derived volatiles are used in conjunction with other chemical cues. In emerald ash borer systems, host- and plant-derived cues interact strongly. *Spathius agrili* was initially found to respond primarily to ash volatiles rather than emerald ash borer larvae or frass (Wang et al. 2010a), whereas later work demonstrated attraction to odor blends integrating host-plant volatiles with emerald ash borer-associated cues (Johnson et al. 2014). Similar multi-cue responses have been documented for parasitoids of *Scolytus multistriatus* (Coleoptera: Curculionidae) and bark beetles in the genera *Ips* and *Dendroctonus*, where parasitoids are attracted to combinations of host-produced and plant-produced compounds (Kennedy 1979, 1984). *Scleroderma guani* (Hymenoptera: Bethylidae), a generalist parasitoid of cerambycid beetles, uses frass and host-altered wood chemicals to guide host-searching for larvae of longhorned beetles such as *Monochamus alternatus* (Li et al. 2015).

#### Microbial Symbiont Cues

Parasitoids attacking concealed hosts are also known to use volatiles produced by microorganisms (bacteria or fungi) associated with the hosts (ie symbionts) or hosts’ environment such as those in decaying fruit or frass of insect galleries (Mills et al. 1991). For example, the ichneumonid wasp *Megarhyssa nortoni*, a biological control agent against the global pine pest *Sirex noctilio* (Hymenoptera: Siricidae), can use chemical information derived from the fungal symbiont–pine complex while foraging for its wood-boring insect host, which engages in an obligate nutritional symbiosis with the fungus *Amylostereum areolatum* (Spradbery 1970, Martínez et al. 2006, Fischbein et al. 2018). Yet, the identity of microbial-related compounds used by *M. nortoni* has not been confirmed. Similarly, Liu et al. (2022) showed that the seasonal flight activity of a braconid parasitoid (*Eucosmophorus* sp.) closely mirrored that of ambrosia beetles in the *Euwallacea fornicatus* complex (Coleoptera: Curculionidae), implicating cues associated with the beetle–fungal symbiosis in parasitoid host location.

Microbial cues rarely act independently; studies suggest that long-range attraction often depends on the combined presence of host insects, fungi, and host plants (Sullivan and Berisford 2004). In bark beetle systems, assemblages of parasitoids and predators have been shown to respond strongly to combinations of *Ips pini* (Coleoptera: Curculionidae), its fungal associates, and inoculated host plants, with some species responding more strongly to microbial cues than to beetle-derived signals alone (Boone et al. 2008, 2012). Unlike insect hosts, microbial associates experience less selective pressure to remain cryptic, making them potentially reliable indicators of host presence (Sullivan and Berisford 2004).

### Vibrational and Other Tactile Cues

For parasitoids attacking concealed hosts, vibrational cues generated by host movement or feeding within the substrate can be critical for short-range host location. Vibrational signals from feeding activity or movement have been documented in a variety of concealed systems; for example, feeding by fruit fly larvae within infested fruit elicits oviposition-probing behavior of 2 opiine fruit fly parasitoids [*Diachasmimorpha longicaudata* and *D. tryoni* (Hymenoptera: Braconidae)] (Duan and Messing 2000). Such cues are thought to be especially relevant for hosts that tunnel deeply into woody tissues, such as buprestids and cerambycids, where chemical cues alone may lack the spatial resolution needed to pinpoint host location (Paine 2017). Both *Syngaster lepidus* and *Callibracon limbatus* (Hymenoptera: Braconidae) locate their hosts using substrate vibrations, with authors noting that the feeding of late instar *Phoracantha semipunctata* (Coleoptera: Cerambycidae) was even audible to the human ear (Hanks et al. 2001). *Doryctes leucogaster* (Hymenoptera: Braconidae), a parasitoid of cerambycids, uses vibrational cues to detect host larval activity within wooden poles (Hilszczański 2018). Even in bark beetle systems, where hosts are closer to the bark surface, vibrational cues alone can induce oviposition behavior. Ryan and Rudinsky (1962) demonstrated this using a pin to scratch the underside of bark, inducing oviposition by *Coeloides brunneri* (Hymenoptera: Braconidae). The use of vibrational cues has been most frequently documented among Braconidae, Eulophidae, and Pteromalidae (Meyhöfer and Casas 1999).

Hymenopteran parasitoids lack tympanic hearing organs, so they rely on substrate vibration to indirectly detect sound (Quicke 1997). Parasitoids using host-generated vibrations have been observed to stop and “listen” while searching. Females may slow or pause movement, contacting the substrate with their antennae before resuming search behavior. For example, *Syngaster lepidus* (Hymenoptera: Braconidae) searched for their cerambycid hosts by slowly and deliberately walking across the bark surface, often stopping and touching their antennae to the bark before resuming (Joyce et al. 2011). *Phymastichus holoholo* (Hymenoptera: Eulophidae), a parasitoid of *Xyleborus* spp. (Coleoptera: Curculionidae), has been observed walking linearly over wood surfaces, arresting at beetle holes or fresh frass, and antennating prior to oviposition, suggesting reliance on close-range tactile and potentially vibrational cues during host assessment (Honsberger and Wright 2022). Morphological support for mechanosensory detection is evident in some parasitoids, such as the EAB larval parasitoid *S. agrili* which has abundant sensilla trichodea on the tibiae (legs) and ovipositor sheaths of female wasps that may be mechanoreceptors and tactile sensors for mechanical or vibration cues (Wang et al. 2010b).

Vibrational cues may also provide information beyond host presence. For example, Paine et al. (2000) suggested that *S. lepidus* and other parasitoids of *P. semipunctata* use vibrational cues to assess bark thickness, a factor which can impact oviposition success. Work on emerald ash borer parasitoids has also shown that emerald ash borer larvae of all sizes produce short, distinct vibrational impulses lasting approximately 3 to 30 ms from their feeding activity within the cambium and phloem. While the rate of impulses does not vary with the larval size, vibration amplitude is significantly higher for larger larvae and is positively correlated with higher progeny production by the larval parasitoid *T. planipennisi*, suggesting that vibrational cues may convey information about host quality to parasitoids of concealed hosts (Ulyshen et al. 2011).

Evidence for the importance of vibrational cues in emerald ash borer systems further illustrates their role in parasitoid host location. Earlier work infers a role for vibration in emerald ash borer host location, noting that *S. agrili* appeared to rely little on semiochemicals (Wang et al. 2010a), or that plant- and host-derived volatiles may primarily stimulate searching behavior, with vibrational cues used to localize hosts at close range (Johnson et al. 2014). Although *T. planipennisi* did not explicitly use vibration to assess host quality in experimental assays, its restriction to actively feeding larvae suggests an indirect reliance on host-generated movement or feeding sounds (Ulyshen et al. 2011). Differences among emerald ash borer parasitoids further support this interpretation: *S. galinae*, an idiobiont parasitoid, does not respond to hosts already parasitized by conspecifics, whereas the koinobiont *T. planipennisi*, whose hosts continue to develop after parasitism, does so, likely because parasitized larvae remain active and continue producing vibrational cues (Wang et al. 2015). Nevertheless, evidence from other concealed-host systems indicates that vibrational cues likely interact with visual and chemical signals rather than functioning in isolation (Duan and Messing 2000, Fischer et al. 2001, 2003), highlighting the need for further research to disentangle the relative contributions of vibrational, chemical, and other cues in host assessment by emerald ash borer parasitoids and parasitoids of concealed hosts more broadly (Hegdekar and Arthur 1973).

Beyond passively detecting host-generated vibrations, some parasitoids actively produce vibrations in a process known as vibrational sounding (Meyhöfer and Casas 1999, Wang and Yang 2008, Wang et al. 2021). This behavior involves generating substrate vibrations, often by tapping with the antennae, and detecting reflected signals (or “echoes”) using specialized sensory subgenual organs located in the tibiae (Broad and Quicke 2000). The use of vibrational sounding is strongly associated with parasitoids that attack deeply concealed hosts, particularly when host depth exceeds the parasitoid’s body size, and is more common among idiobionts (Broad and Quicke 2000). For example, Samson (1984) found that *Roptrocerus xylophagorum* (Hymenoptera: Pteromalidae), a parasitoid of *Ips* which enters beetle galleries to oviposit, seems to locate its hosts by tapping its antennae on the sides of host galleries. Most studies on this tactic have focused on parasitoids of concealed but not wood-boring hosts, such as *Pimpla turionellae* (Hymenoptera: Ichenumonidae), a pupal parasitoid of a pyralid moth that uses tibial receptor organs to detect self-produced vibrations (Otten et al. 2002). The wasp was able to compare vibrations detected via each of its 3 pairs of legs to determine the precise position of hosts. Despite there being less focus on parasitoids of wood-boring beetles, a comparative morphological study of ichneumonid parasitoids demonstrated a significant association between the degree of antennal hammer development, an adaption used for vibrational sounding, and parasitism of wood-boring hosts such as Cerambycidae and Buprestidae, suggesting that vibrational detection is an evolutionary adaptation favored in parasitoids attacking hosts within wood (Laurenne et al. 2009).

While there is substantial evidence supporting the role of vibration, not all parasitoids appear to use vibrational cues. Some bark beetle parasitoids probed bark indiscriminately regardless of host presence, suggesting vibration was not involved in host detection (Mills et al. 1991). Other studies failed to detect sounds produced by chewing larvae or parasitoids themselves (Richerson and Borden 1972a), and some parasitoids readily oviposit on dead hosts, indicating that host-generated vibrations are not always required (Spradbery 1970, Wegensteiner et al. 2015). Additionally, the effectiveness of vibrational sounding may decline in dense substrates, potentially limiting its utility in some wood-boring systems (Fischer et al. 2003).

Other tactile or physical cues of the hosts’ microhabitat (eg bark roughness of infested host trees) may also play an important role in host finding by egg parasitoids of wood-boring beetles. For example, gravid emerald ash borer females select oviposition sites based on the size and tightness of bark crevices once a suitable ash host is located (Green and Duan 2025). These bark attributes may, in turn, influence the host finding efficacy and thus rate of emerald ash borer eggs parasitized by the introduced biocontrol agent *Oobius agrili* (Hymenoptera: Encyrtidae) (Jennings et al. 2018). In addition, some parasitoids thread their ovipositor through tunnels made by their hosts, suggesting that they “feel” for their hosts rather than detecting individuals from the bark surface (Wang et al. 2021).

### Visual Cues

Visual information, particularly color, can be an important stimulus to host-finding at long distances by parasitoids of both concealed and exposed hosts (Morehead and Feener 2000, Castillo and Rojas 2020). Although parasitoid hymenopterans generally possess smaller compound eyes relative to many nonparasitic hymenopterans and are considered to rely predominantly on chemical cues, they exhibit trichromatic vision with photoreceptors sensitive to ultraviolet, blue, and green wavelengths (Desouhant et al. 2010, Fischer et al. 2011, Lim and Ben-Yakir 2020). This visual capacity enables discrimination of color and contrast that may contribute to habitat orientation and host-associated searching.

Many parasitoids are highly attracted to yellow visual cues, which broadly overlap with the reflectance range of green foliage, and may be attributed to the visual signal of pollen (Gates et al. 1965, Prokopy and Owens 1983, Wäckers 1994). Thus, yellow sticky cards and yellow pan traps have been used for effective parasitoid sampling in crop fields or forests. For example, yellow pan traps installed on ash trees are useful in sampling the relative abundance of both introduced and native North American egg and larval parasitoids associated with emerald ash borer and other wood-boring beetles in the United States (Petrice et al. 2023). In addition, colors associated with host macrohabitats, including blue, purple, and red hues, have been shown to attract parasitoids attacking concealed hosts. For example, the purple prism trap designed for trapping adult emerald ash borer is also attractive to several species of native wood borer larval parasitoids such as *Phasgonophora sulcata* (Hymenoptera: Chalcididae) and *Atanycolus* spp. (Hymenoptera: Braconidae) that also attack emerald ash borer larvae (Gaudon et al. 2020). It is also important to note that visual cues associated with the hosts’ habitats can interact with chemical cues of the hosts’ habitats and or hosts themselves to aid in the host finding by these parasitoids (Fischer et al. 2001).

Aside from color, other visual cues may contribute to host searching by parasitoids of wood-boring insects. Although direct evidence for long-range “silhouette” orientation in these parasitoids is limited, visual features on the bark surface can guide close-range searching. For example, the parasitoid of *Xyleborus* spp., *P. hololo* slows or stops at beetle entrance holes or fresh frass prior to antennation, suggesting that short-range visual cues are used as it explores the wood surface (Honsberger and Wright 2022). Similarly, *Sclerodermus guani* (Hymenoptera: Bethylidae), which primarily attacks cerambycids and buprestids, uses host-produced frass visible on the bark surface as a visual cue to locate host galleries before using chemical cues within the galleries to ultimately detect the host (Li et al. 2015).

### Infrared Cues

Although primary cues for hymenopteran parasitoid host finding are typically chemical (olfactory), mechanical (vibrational), or visual, some species have been hypothesized to use infrared radiation to locate concealed hosts. Richerson and Bordon (1972a,b) and Richerson et al. (1972) suggested that *Coeloides brunneri* (Hymenoptera: Braconidae), a parasitoid of subcortical bark beetles, jewel beetles, and longhorned beetles in conifers, could locate and oviposit on host larvae by detecting heat radiation emitted from host metabolic activity. Supporting this hypothesis, oviposition was induced when the underside of bark was artificially heated using wire probes. Subsequent morphological work identified sensilla on the antennae of some wasps within Ichneumonoidea that had morphological structures hypothesized to detect infrared heat radiation (Borden et al. 1978). However, attempts to identify host-associated thermal “hotspots” in other parasitoid systems have yielded mixed results. For example, Mills et al. (1991) and Pettersson et al. (2001b) investigated whether scolytine parasitoids such as *Coeloides bostrichorum* (Hymenoptera: Braconidae) and *Rhopalicus tutela* (Hymenoptera: Pteromalidae), respectively, could use localized temperature differences to locate hosts beneath the bark, but neither study detected consistent thermal cues associated with host presence. In addition to metabolic heat, host movement and feeding activity within substrates may generate localized thermal signatures that parasitoids could exploit as short-range directional cues. However, it is largely unknown whether the minute amounts of infrared radiation produced by host metabolism or movement are sufficient to play a meaningful role in host detection for parasitoids. Moreover, it is generally assumed that the effectiveness of infrared cues is likely constrained by environmental factors, such as heterogeneity in bark temperature caused by light and shadow, which can create a patchwork of thermal variation that obscures any host-specific infrared signal (Quicke 1997, Meyhöfer and Casas 1999).

## Learning to Use Cues for Host Habitat and Host Finding

Experience and learning can enable parasitoids to associate chemical cues with location of their hosts (eg Giunti et al. 2015). While detection of host-derived chemical cues is typically innate, parasitoids can learn to associate novel environmental cues with host presence, thereby overcoming the reliability–detectability dilemma (Vet et al. 1995). For parasitoids attacking wood-boring insects, which typically exploit larval hosts, learning may be particularly advantageous because host-associated cues tend to remain consistent within an individual parasitoid’s lifetime, even if they vary across generations (Vet et al. 1995). Learning serves to both increase the response to stimulus and decrease the variability of the response (Vet et al. 1990).

For example, a parasitoid of Drosophila flies, *Leptopilina heterotoma* (Hymenoptera: Figitidae), learned to respond to a novel odor and use that odor in habitat selection, with oviposition experience reinforcing this learning (Vet and Groenewold 1990). While few studies focusing on parasitoids of wood borers have explored the topic, some have evaluated the role of learning. For instance, naïve *Sclerodermus guani* (Hymenoptera: Bethylidae) did not respond to odor sources from cerambycid larval habitat, whereas females exposed to sawdust odors from infested trees responded to these cues (Li et al. 2015). Similarly, *Sclerodermus pupariae* (Hymenoptera: Bethylidae) exposed to cues of novel hosts [host plant bark and frass of emerald ash borer and *Massicus raddei* (Coleoptera: Cerambycidae)] not only preferred odors associated with the host they were previously exposed to, but they were also more efficient at parasitizing those hosts (Wei et al. 2013b).

Learning of different host or host habitat-associated cues may also occur among populations within a single parasitoid species. For example, the coleopteran parasitoid *Dastarcus helophoroides* (Coleoptera: Bothrideridae) responds to different frass-derived volatile kairomones associated with different host species: Populations attacking Asian longhorned beetle are attracted to (S)-(–)-limonene, whereas those attacking *Apriona swainsoni* (Coleoptera: Cerambycidae) respond primarily to α-pinene and β-pinene isomers (Wei et al. 2013a). As demonstrated in many studies, learning typically occurs during the adult stage, and preadult learning is very rare (Vet and Dicke 1992). The ability of parasitoids to learn host-associated cues raises the possibility of conditioning individuals prior to release by exposing them to volatiles from target hosts or infested substrates during rearing. Such pre-release experience could increase responsiveness to specific host cues, enhance search efficiency in the field, and ultimately improve biological control efficacy against wood-boring pests.

Although learning may allow parasitoids to detect novel hosts, host finding alone does not guarantee biological control success, as host quality and behaviors can influence parasitoid performance. For example, *D. helophoroides* can locate and parasitize both its native host Asian longhorned beetle and a nontarget surrogate emerald ash borer, yet parasitism success, development time, and offspring size are significantly reduced on emerald ash borer, likely reflecting differences in host quality and emerald ash borer’s frass-packing behavior (Rim et al. 2018).

## Implications for Biological Control Programs

The success of biological control often hinges on efficient host foraging by the concerned parasitoid agents for successful location of the targeted pest hosts for reproduction. Understanding the behavioral or ecological mechanisms and various cues used in the process helps practitioners select efficient, host-specific agents for successful biocontrol of the targeted pests. For example, a major obstacle to biological control of wood-boring insects is the restriction imposed by a parasitoid’s ovipositor length and its capacity to penetrate different bark thicknesses, which directly constrains both host access and effective host finding. Abell et al. (2012) found that *T. planipennisi*, a species used in the emerald ash borer classical biological control program with an ovipositor length of 2.0 to 2.5 mm (Duan and Oppel 2012), cannot parasitize emerald ash borer in trees with bark thicker than 3.2 mm. To overcome the disadvantage of *T. planipennisi* in protecting larger American ash trees, another braconid wasp with a much longer ovipositor (4 to 5.3 mm), *S. galinae*, was later introduced to North America for emerald ash borer biocontrol. This latter introduced agent can reach emerald ash borer larvae in larger ash trees with thicker bark (beyond 6.5 mm) and thus has played an important role in protecting large ash trees against the invasive beetle(Murphy et al. 2017, Duan et al. 2023b). In addition, *Atanycolus* spp., a genus of native emerald ash borer parasitoids with an ovipositor length of 4 to 6 mm (Marsh et al. 2009), can parasitize beetles in trees with bark thickness up to at least 8.8 mm (bark thicker than 8.8 mm was not part of the study). Although ovipositor length presents a limitation, the ability of these parasitoids to parasitize emerald ash borer in trees with an average bark thickness greater than their ovipositor lengths suggests that host finding includes the capacity to locate thinner or more penetrable areas of bark. Similarly, Lupu et al. (2025) detected successful parasitism of Asian longhorned beetle by *Ontsira mellipes* (Hymenoptera: Braconidae), but only on small-diameter branches with thinner bark. The authors suggest this could reflect the limitations imposed by the parasitoid’s relatively short ovipositor (mean length 3.91 mm) (Wang et al. 2019, Lupu et al. 2025). Consequently, ovipositor length relative to host tree bark thickness has important implications for biological control success, as released agents must be able not only to locate hosts but also to physically access them within the size classes of trees present in the stand.

In addition, identification of cues used in host-foraging or finding can improve the design or development of tools for monitoring the establishment, abundance and/or impact of the introduced or released parasitoids on the target pests. For example, yellow pan traps are effective at capturing many hymenopteran parasitoids which are attracted to yellow color associated with their hosts’ food plants or trees. However, traps such as yellow pans relying on generic visual cues lack the specificity or selectivity in sampling parasitoids introduced for biocontrol, and their efficacy also depends on the appropriate installation on host-infested trees or habitats, implicating the importance of other habitat or tree-associated cues in attracting foraging parasitoids. More recently, a field study comparing detection techniques for introduced emerald ash borer larval parasitoids found that sentinel ash logs infested with emerald ash borer larvae detected parasitism by *T. planipennisi* and *S. galinae* more frequently than yellow pan traps (Rutledge et al. 2021). However, the relative effectiveness of these monitoring approaches depends on factors such as deployment location, seasonal timing, and environmental conditions. Nevertheless, the higher detection rates observed with sentinel logs suggest that cues associated with infested host material and hosts themselves can play an important role in attracting foraging parasitoids.

Although sentinel logs artificially infested with emerald ash borer or ALB provide a practical and standardized tool for post-release monitoring, their effectiveness may be constrained by the host-finding cues relied upon by parasitoids. Because sentinel logs are cut from living trees, they likely emit volatile profiles that differ from those of intact, stressed, or actively infested hosts, potentially reducing their attractiveness to foraging parasitoids that use host- or plant-derived semiochemicals at long range. In addition, important short-range cues such as frass, feeding vibrations, or induced plant responses may be weak or absent when logs are deployed before host larval activity produces these cues. As a result, parasitoids may fail to locate or respond to sentinel logs even when established in the surrounding environment, leading to underestimation of establishment or activity. These limitations highlight the need to interpret sentinel log data cautiously and, where possible, to complement them with additional sampling methods that better reflect the full suite of cues used during host location.

## Conclusion and Future Directions

The use of parasitoids for biological control is immensely important for the management of wood-boring beetle pests. Some parasitoids as biocontrol agents have played a pivotal role in the management of invasive wood-boring beetles, such as EAB (Duan et al. 2023a, Gould et al. 2024). Relatively little research has been done on the mechanisms used by these parasitoids to locate or find their highly concealed hosts. Host finding is a multistep process that integrates diverse cues from the host, its habitat, and associated symbionts and microorganisms, all of which are critical for effective biological control. Unfortunately, few studies have examined the specific mechanisms and cues used in host finding by parasitoids of wood-boring hosts over complex and patchy environments. Researching this field along with the role and potential use of associative learning by parasitoids in finding wood-boring beetles could greatly enhance biological control efforts. Additionally, research into associative learning and conditioning of these parasitoids could provide pathways to facilitating the use of novel hosts for biological control (eg Golec et al. 2019).
